# The Role of Different Types of Actors In The Future of Sustainable Agriculture In a Dutch Peri-urban Area

**DOI:** 10.1007/s00267-022-01654-3

**Published:** 2022-05-04

**Authors:** Catharina J. E. Schulp, Franziska Komossa, Laura Scherer, Emma H. van der Zanden, Marta Debolini, Annette Piorr

**Affiliations:** 1 Institute for Environmental Studies, Environmental Geography Group, Vrije Universiteit Amsterdam, De Boelelaan 1111, 1081HV Amsterdam, the Netherlands; 2 Institute of Environmental Sciences (CML), Leiden University, Einsteinweg 2, 2333 CC Leiden, the Netherlands; 3 PBL Netherlands Environmental Assessment Agency, Bezuidenhoutseweg 30, 2594 AV The Hague, the Netherlands; 4 UMR EMMAH INRAE/AU, 228 route de l’Aérodrome, 89914 Avignon, France; 5 Leibniz Centre for Agricultural Landscape Research (ZALF) e.V., 15374 Müncheberg, Germany

**Keywords:** Bayesian Belief Network, Sustainable Intensification, the Netherlands, Local supply chains, Regional agri-food system, Stakeholder participation

## Abstract

Peri-urban areas support a broad range of multifunctional demands for public goods. In northwest Europe, peri-urban areas tend to overlap with intensive agricultural land, resulting in conflicts between agricultural use and the public good demands of residents. Sustainable intensification (SI) of agriculture might help reconcile agricultural and well-being goals, but it is unclear how the mix of actors in a peri-urban setting can trigger or restrain SI. In a Dutch case study, we explored how SI of agriculture can contribute to making peri-urban areas more sustainable, and which actors are key enabling factors for implementing SI. We used interviews, surveys, workshops, and empirical analysis to obtain insight into the stakeholder’s vision of a sustainable future for the case study area, the farming system and actor network. We integrated these insights in a Bayesian Belief Network, where we linked the actor network to implementation of three SI measures (farm-level efficiency measures, small landscape elements, and direct sales), and used sensitivity analysis to model effects of support for implementation by different groups of actors. The case study has a dense stakeholder network, where, dependent on the SI measure, farmers are triggered by all actors to implement SI, or have a stronger role in uptake themselves. The sensitivity analysis suggested that the future preferred by the stakeholders requires broad support of all actors involved, with local actors without a formal role being essential for uptake. Overall, trade-offs among public goods are almost inevitable when taking up SI measures.

## Introduction

Across Europe, 79% of the population lives in urban or peri-urban areas (Eurostat 2021). The population of urban agglomerations is increasing, primarily on the fringes around medium-sized cities in Western Europe (Kabisch and Haase 2011). This peri-urbanization process combines built-up area expansion, farmland loss and fragmentation, and restructuring of the agricultural sector, primarily toward a more diverse source of income (Shaw et al. 2020; Spyra et al. 2020). Future population and socio-economic projections suggest further peri-urbanization throughout Europe (Shaw et al. 2020).

The combination of residential and agricultural land use in peri-urban areas supports many public goods (non-excludable, non-rival goods and services that are beneficial to society) and other ecosystem services (benefits to humans provided by ecosystems) (Knickel and Maréchal 2018; Shaw et al. 2020; Termorshuizen and Opdam 2009). Peri-urban areas are, for example, important for outdoor recreation (Komossa et al. 2020), and for the local urban food system (Jansma and Wertheim-Heck 2021; Zasada et al. 2019). The multifunctionality of peri-urban landscapes supports the demands that many young Europeans express about their living environment: an environment that produces local, ecologically friendly food, gives access to green infrastructure, job opportunities at close commuting distance, and space for a private garden (Frei et al. 2020; Metzger et al. 2017; Rudel 2020; Sylla et al. 2020).

The combination of residential and agricultural land use in a limited space also results in conflicts and increasing land prices. In about 20% of peri-urban areas, continued intensification occurs (Shaw et al. 2020), which is often characterized by land consumption alongside intensified agricultural production (Spyra et al. 2020). Conflicts emerge, among others, regarding water quality, biodiversity (Ladrera et al. 2019; Marmonier et al. 2018), and human health impacts due to, among others, zoonoses and particulate matter emissions (Linhart et al. 2019; Post et al. 2020). Furthermore, peri-urbanization reduces open space, threatening the supply of ecosystem services (Spyra et al. 2021), and intensification conflicts with landscape preferences of urbanites, who appreciate peri-urban areas for recreation in their accessible nature and traditional farming landscapes (Almeida et al. 2016; Tieskens et al. 2018). A need to ensure farm viability (Almeida et al. 2016) combined with increasing land prices triggers specialization toward systems with high area profitability such as horticulture or service orientation like “horsification” (Zasada 2011). The mix of land uses and users in peri-urban areas is challenging to govern, as public interventions are split over different policy domains (Doernberg et al. 2019; Spyra et al. 2020), while individual farm management choices can also have strong impacts (Shaw et al. 2020).

The prevalence and expected increase of peri-urban areas in Europe call for a transition toward more sustainable farming systems, that allow to accommodate the needs of different users. Vermunt et al. (2022), for example, studied the transition to nature-inclusive farming but identified, among others, the lack of a shared vision for nature-inclusive farming as well as the lack of specific and integral knowledge as key blocking mechanisms for its uptake. Agroecology, a holistic approach toward food system design and implementation (Bezner Kerr et al. 2021; Runhaar 2021), and sustainable intensification (SI), which can be defined as increasing or levelling production while reducing negative impacts on the environment and society (Godfray and Garnett 2014), face similar conceptual unclarity and challenges related to uptake (Armstrong McKay et al. 2018; Runhaar 2021). Nevertheless, SI, in a broad sense, addresses the variety of services demanded in peri-urban areas (Weltin et al. 2018): it aims at ensuring farm viability through levelling production, while also decreasing pressures on the environment and accommodating recreation. Taking a governance strategy perspective, Rudel (2020) recently identified SI pathways for peri-urban areas, where local food chains and agro-biodiversity play an important role. However, the potential of SI is underexplored in peri-urban areas (Weltin et al. 2018) and in particular, there is little insight into how the challenging governance setting inherent to peri-urban areas (Spyra et al. 2020) interacts with farm-level drivers for decision-making on adopting SI, such as the level of farmers’ professionalization (Albizua et al. 2020; Häfner and Piorr 2020; Knierim et al. 2017). Taking a farm-level perspective, the adoption behavior of SI measures by farmers is an extensively researched field, identifying among farm specialization, structural, and socio-demographic factors (Greiner 2015; Meraner et al. 2015) also behavioral factors like social norms (Dessart et al. 2019). Following Dessart et al. (2019) and investigating dispositional, social, and cognitive factors that play a role in the beliefs, motivations, attitudes and networks of a farmer, Weltin et al. (2021) distinguish different pathways of SI portfolio broadening based on empirical data and modelling. The intention for additional uptake of regional marketing was, for example, positively related to farmers attitudes toward economic and environmental sustainability (dispositional factors), while the intention to adopt precision farming or establishing new landscape elements was mainly driven by experience (cognitive factors). The significance of social interaction constructs, i.e., frequency and value of interaction and networking with other actors, could not be proven due to high data variance in that particular study. However, it is precisely the different quality of peri-urban settings and processes that leads us to assume a knowledge gap, which we want to address with a multi-method approach.

In this study, we aim to quantify how the variety of actors relevant in a peri-urban setting interacts with explanatory factors at the farm level that influence decision-making on adopting SI. We focus on a single peri-urban case study in the Netherlands to allow an in-depth exploration of this under-researched setting for SI. We use a mixed-methods approach, where we combine heterogeneous empirical data from a peri-urban case study in the Netherlands in a Bayesian Belief Network (BBN) to explore which actors or actor groups stimulate or block uptake of more sustainable agricultural practices.

## Background

### Theoretical Background

#### Sustainable intensification

While some definitions of SI strictly focus on reducing trade-offs between production and environment, we adopt a broader definition that also includes the human dimension of sustainability through farm viability and food security, access, and distribution (Thomson et al. 2019). Weltin et al. (2018) conceptualize SI according to the spatial scale of intervention (farm or region) and the effect of interventions on either land use or the agricultural system. This conceptualization groups SI measures in four “fields of action” (FoA’s): Farm-level land use (FoA1) and system (FoA2) measures, and regional land use (FoA3) and system (FoA4) measures. This broader definition of SI supports balancing the multiple demands in peri-urban areas. Farm-level land use measures (FoA1) include e.g., tree lines that limit pesticide drift, while farm-level system measures (FoA2) include e.g., filters that reduce ammonia and odor emission from stables. Landscape planning at a regional scale (FoA3) can support the high peri-urban recreation demands. Diversification toward a stronger local food system and supply chain (FoA4) can foster economic viability and local food security (Güneralp et al. 2020).

#### Uptake of SI

**Fig. 1:**
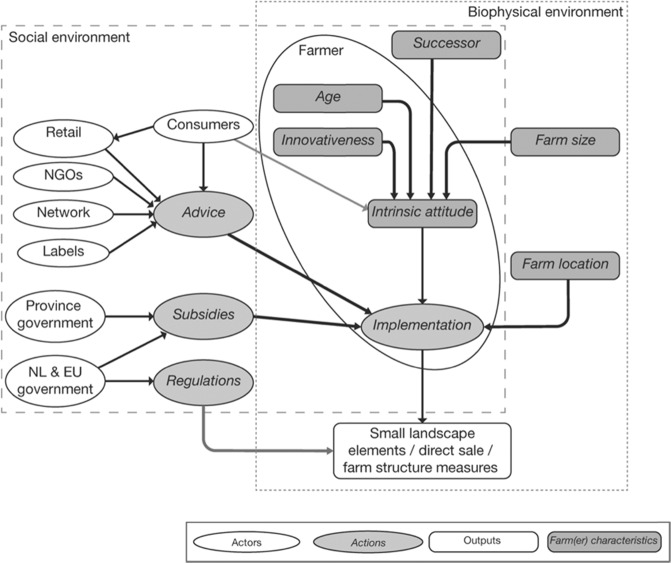
Conceptualization of actors and environment for uptake of SI measures

We base our contextualization of farmer behavior on Schlüter et al. (2017) who evaluate a wide range of theories and frameworks that describe human decision-making about natural resources, generalizing them into a framework (MoHub) that facilitates including the understanding of human decision-making into environmental modelling (Fig. 1). The MoHub framework distinguishes a *biophysical* and *social* environment that provides context for, and influences, decision-making of the individual. Within the MoHub framework, we contextualize farmer behavior according to the Theory of Planned Behavior (Ajzen 1991), because of its consideration of beliefs about the effect of behavior and their normative value, providing a means for including the impact of the wide range of actors in a peri-urban setting that influence farmers’ beliefs. The Theory of Planned Behavior suggests that implementation of measures results from an *intention*, underpinned by an *attitude* toward the behavior, *subjective norms*, and *perceived behavioral control* (Ajzen 1991). These different aspects are elements of the social and biophysical environment that influence farmer’s behavior, and specify the role of *assets*, *resources*, and *opportunities* for explaining behavior. Research shows that farmer’s *attitude* originates from intrinsic motivation (Runhaar 2017). For example, empirical studies in Germany suggest that fostering pro-environmental behavior or feelings of responsibility could positively impact adoption of SI measures (Weltin and Hüttel 2019; Weltin et al. 2021). *Perceived behavioral control* is contextualized as the social network of farmers (Schlüter et al. 2017), which includes other farmers in informal networks (Albizua et al. 2020; Barnes 2016; Garbach and Morgan 2017), extension services (Ingram and Mills 2018; Knierim et al. 2017), and market actors such as retailers, wholesalers, or processors (sometimes strengthened by voluntary standards (Smith et al. 2019)).

The MoHub framework distinguishes a *biophysical* and *social* environment (Fig. 1). *Biophysical* environmental conditions can influence farmers’ actions (Hijbeek et al. 2017; Mouysset 2016), together with assets such as farm size (Pavlis et al. 2016). The *social* environment consists of the social network, as well as the policy and regulatory context that includes among others policies such as the Common Agricultural Policy, including greening regulations (Cortignani and Dono 2018), the Water Framework Directive (Biernat et al. 2020), and voluntary-based interventions (Barnes 2016).

### Case Study

**Fig. 2:**
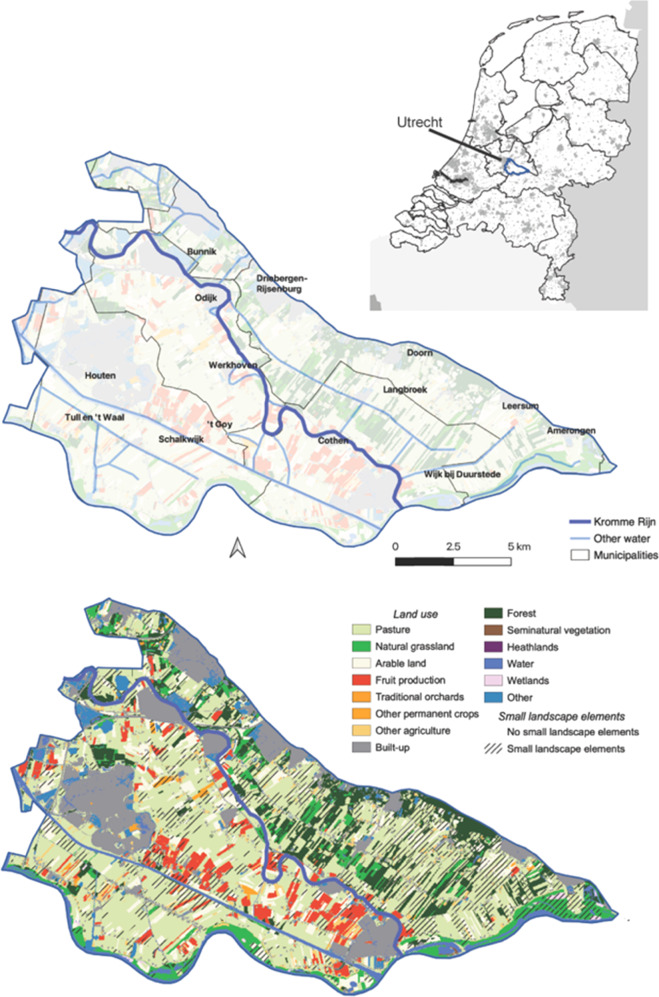
Location, basic topography, and land use of the Kromme Rijn case study area

The Kromme Rijn area (Fig. 2) is adjacent to Utrecht, the fourth largest city of the Netherlands. It is an important recreation area essential to the well-being of Utrecht’s citizens (Provincie Utrecht 2010), as well as to the more than 138,000 inhabitants of the case study area (CBS 2020). Land use in the 220 km^2^ case study area is a mix of large, dense villages, dispersed houses, farms and farmland, and nature areas. Agriculture is dominated by dairy and fruit production: among its 382 farms are 284 dairy farms, 71 fruit growers, and 27 arable farms. Given the dominance of dairy and fruit production in the landscape and agricultural sector, we focus our analyses on these two sectors. Southwest of the Kromme Rijn river (Fig. 2) larger farms of higher intensity are found; the north-eastern part has a mixed landscape structure and farming tends to be less intensive. The case study area is a typical example of a peri-urban landscape with intensive agriculture in the Atlantic region of Europe (van der Zanden et al. 2016), and also a typical example of a fast-growing urban fringe region (Kabisch and Haase 2011), making it a relevant example of a widespread ongoing land use change process (Shaw et al. 2020).

The case study area covers parts of four municipalities, all within Utrecht province. Municipalities develop and implement spatial planning of built-up areas, and develop municipality level energy strategies. The province is responsible for all policies on nature, agriculture, spatial planning, environment and rural development (Nitsch et al. 2017). In the Netherlands, only farmer collectives can be beneficiaries of agri-environmental subsidies since 2016 and therefore, agri-environmental measures are organized and implemented by a collective of landowners (Eichhorn et al. 2020). The water board Stichtse Rijnlanden is responsible for water quality and quantity management. This split of responsibilities over different governance bodies is exemplary for the complex governance of peri-urban areas.

## Methods

### Overview

**Fig. 3:**
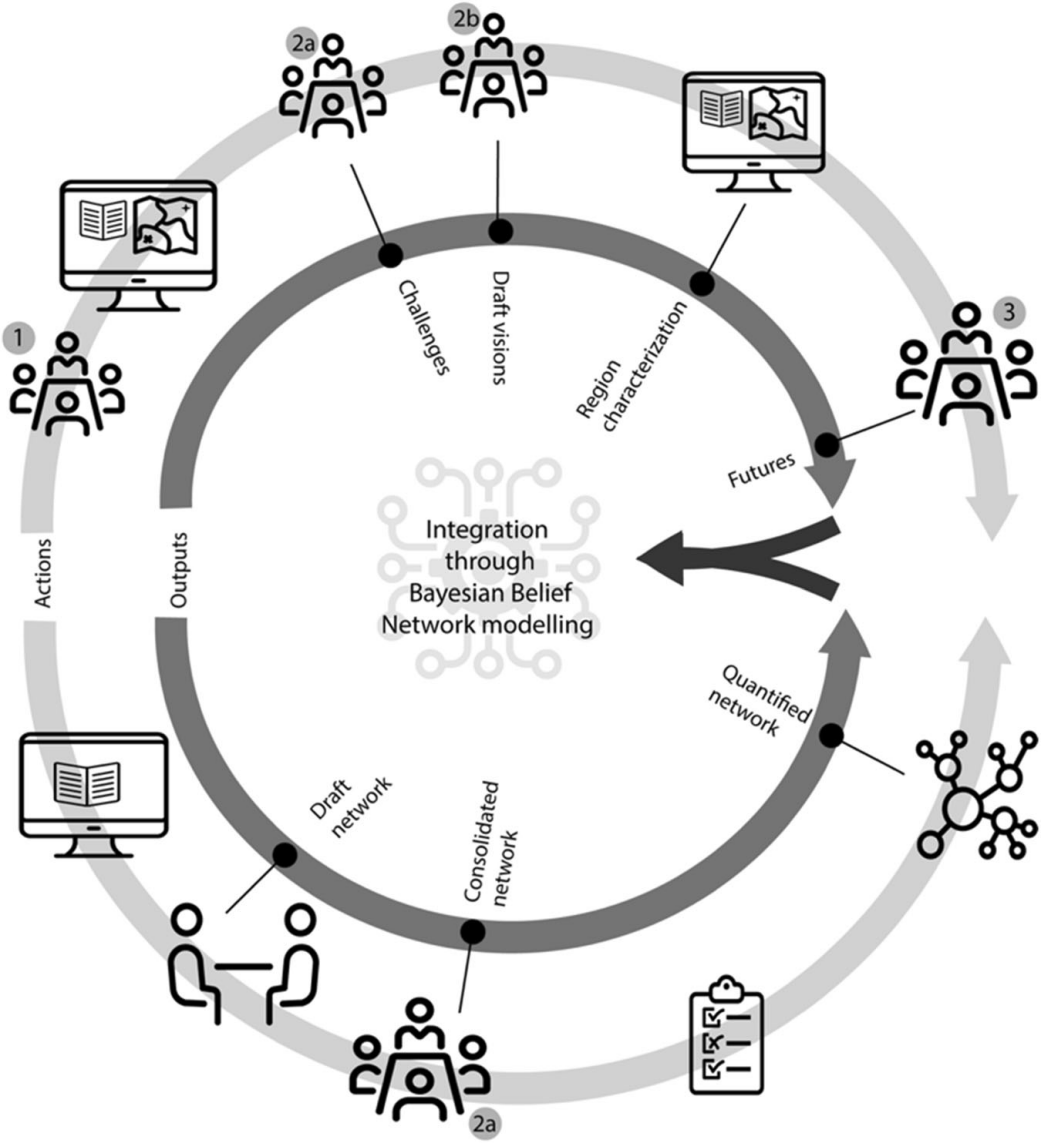
Overview of the methodology. Numbers refer to specific workshops (section *Workshops*). Icons from thenounproject.com under Creative Commons CCBY license

Understanding the agronomic and socio-economic background of the case study area and the potential for future SI required data from different domains. We combined different methods to integrate quantitative data on the potential of SI with qualitative data on the role of actors (triangulation; (Olsen 2004)) (Fig. 3).

To understand actor interactions, we mapped and analyzed the stakeholder network (section *Interviews*). Following Hersperger et al. (2010), we use the term “actor” when addressing “the decisions of people and institutions that execute these actions” and, following Spyra et al. (2018), we use the term “stakeholder” when addressing those “having a particular interest as they represent a community or group interest”.

To get insight into the potential for and impact of SI, we collected and analyzed data on farm structure and land cover, and used workshops to highlight the challenges and desired future solutions to the case study area (section *Workshops*, *Surveys*).

In a BBN (Section *Set-up of the Bayesian Belief Network*), we integrated the role of different actors with the potential uptake of SI measures to evaluate landscape consequences, and simulated different scenarios of support or lack thereof by different actors. This approach was chosen because BBNs are common tools to model complex systems, as they allow combining qualitative and quantitative data (Salliou et al. 2017), are suitable tools for modelling systems where empirical data are scarce (Roberton et al. 2021), and allow combining bottom-up and top-down actions, as well as quantitative scenario development and evaluation (Mallampalli et al. 2016).

### Stakeholder Interaction

#### Interviews

Interviews conducted in July–December 2016 with local and supply chain actors and stakeholders informed a stakeholder network analysis and BBN parameterization, and provided baseline information on the challenges the case study area is facing. A preliminary actor inventory was done based on a web inventory, after which a first relevant stakeholder was contacted who, based on their professional profile, had the best overview of the issues at hand in the case study area. Subsequently, we followed a snowball approach where respondents were asked to identify other important actors. We continued snowballing until saturation and ensured to cover at least the preliminary actor inventory. The interviews were semi-structured, to ensure consistent coverage of all topics. After an introduction, respondents were asked to freely list challenges to the agricultural sector and the case study area. Next, respondents were asked to indicate who influences decision-making on improving the sustainability of farming in general. Specific SI measures were addressed, dependent on the interviewee, where e.g., landscape management was emphasized in interviews with farmers, while supply chain developments were discussed in an interview with a retail organization. Twelve interviews were conducted, with farmers, the dairy sector organization, a retail organization, landscape management and agricultural organizations, government representatives from the water board and the province, experts on agricultural biodiversity, and representatives of the dairy and fruit production sectors. Interviews lasted 1–2 h, were transcribed during the interview and elaborated directly afterward.

#### Workshops

We organized three workshops in the case study area, that aimed to get insight into the challenges to the case study and visions on the future, and to consolidate understanding of the actor context. The workshops contributed to European research projects TALE (https://www.ufz.de/tale/), VITAL (http://vital.environmentalgeography.nl), and CONSOLE (https://console-project.eu) by informing follow-up research and the consecutive workshops.

Workshop 1 (Fig. 3) in March 2016 aimed to get acquainted with the case study area and the stakeholders, and to make an inventory of the spatial planning challenges the case study is facing (Hagemann et al. 2020; Verhagen et al. 2018).

The second workshop in December 2016 consisted of two distinct parts. Workshop 2a (Fig. 3) addressed the agricultural sector and aimed to specify the challenges inventoried in workshop 1 into a common description of the status quo regarding SI. This was done through structuring challenges to the case study area according to the conceptual framework of SI from Weltin et al. (2018). The workshop also aimed to sketch a business-as-usual scenario. For this, participants free-listed SI measures in place in the case study area, and explored the potential of SI measures to deal with regional challenges under a business-as-usual scenario, through a roundtable discussion (Weltin et al. 2018). The third aim of the workshop was to validate a draft stakeholder network map that emerged from the interviews. Workshop 2b (Fig. 3) focused on the landscape and aimed to develop scenarios for landscape development. We used land sharing vs. land sparing as a basis for scenario development, to disclose visions on the future of the landscape (Hagemann et al. 2020). After introducing the concepts of land sharing and sparing, participants were asked to deliberate how much a set of landscape and farm system indicators would change under the assumption that the whole case study area would adopt land sharing or land sparing. In a participatory mapping exercise, the participants indicated where and how the landscape might change in these contrasting land use configurations (Karner et al. 2019). Building on these exercises, the desired future landscape was discussed.

Workshop 3 in March 2018 aimed to create integrated future scenarios of the landscape and the agricultural sector. After a presentation of a literature and data inventory of external challenges to the case study area, as well as the business-as-usual scenario and landscape development scenarios from workshop 2, workshop participants sketched local responses to these challenges, specified the role of actors, and discussed a preferred future for the case study area. This was done following the approach of combining top-down and bottom-up scenario development described by Nilsson et al. (2017).

The workshops hosted between 8 and 14 participants (see supplementary material 1). Based on the stakeholder overview (section *Interviews*), we actively invited at least one participant per group of stakeholders, resulting in a balanced representation of the groups and a broad range of views.

#### Surveys

We gauged consumers’ interest in local supply chains (FoA4, see section *Background*), using a survey on recreational preferences of outdoor recreationists in the case study area among 201 people (Komossa et al. 2019). The survey included six questions on purchasing farm products at the farm gate. We inventoried which products consumers purchased, at which outlets, and expenditures and motives for buying directly at the farm. Furthermore, a survey in the context of the VITAL project (Weltin and Zasada 2018) among farmers in four European countries provided insight into the motives of farmers regarding the uptake of different categories of SI measures and their relations with socio-economic indicators. The data provided insights that were triangulated with workshop and interview (section *Interviews*, *Workshops*) data.

### Social Network Analysis

**Fig. 4:**
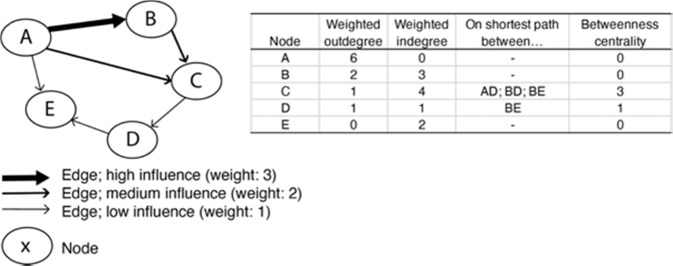
Illustration of social network indicators

We mapped and quantified the relationships and flows between different actors (Martino and Spoto 2006; Prell et al. 2009). The interviews (section *Interviews*) were coded according to how interviewees rated the importance of different actors in their own decision-making as well as for the decision-making of others. Following the inventory of local relevant issues, the social network analysis focused on the decision-making with regards to taking up direct sales (FoA4), measures targeted at supporting landscape diversity by establishing/maintaining small landscape elements (FoA1/3), and farm-level measures that increase efficiency (FoA2) such as modifying crop protection practices in fruit or grazing schedules in dairy. In the interviews, we inventoried the question “how does actor x influence actor y’s decision with regard to…”. Ways of influence were classified as no influence, low influence (e.g., an advisory role), high influence (e.g., financial dependency), or mandatory. The classifications of influence were summarized in a matrix and checked in workshop 2, which led to minor modifications. Next, a social network analysis was performed in Gephi (Bastian et al. 2009). To obtain insight into the place of different actors in the network, we calculated three indicators (Fig. 4): the number of incoming edges (indegree) and outgoing edges (outdegree) weighted by the strength of the influence reflect the influence that is experienced or exerted by an actor. The number of times a node is on the shortest path between two other nodes (betweenness centrality) reflects the relevance of an actor as a bridge between other actors (Fliervoet et al. 2016; Prell et al. 2009).

### Set-up of the Bayesian Belief Network

#### Overview

A BBN was constructed in GeNie, a Bayesian network inference engine with a graphical user interface (BayesFusion 2020). BBNs are probabilistic models that compute inference through a causal model (Roberton et al. 2021). The BBN was used to explore the role of different actors on a selection of SI measures (maintenance or establishment of small landscape elements (FoA1/3), efficiency measures such as more efficient crop protection application or low-emission stables (FoA2), and direct sales (FoA4)).

We used the conceptual framework (Fig. 1) and the stakeholder network (section *Social Network Analysis*) as a basis for the BBN. All actors, actions, farm or farmer characteristics, and outputs were implemented as nodes, where the decision to adopt a measure or not was a node (Fig. 4) that was informed by a decision of a farmer. We constructed BBNs for the fruit and dairy sectors separately, as stakeholders suggest different dynamics in the dairy and fruit supply chains. Linkages and conditional probability tables were further specified based on the stakeholder interaction (section *Stakeholder interaction*) and auxiliary data (Supplementary material 2). A preliminary version of the BBN was verified by two stakeholders who attended all workshops, and a local expert. This led to minor modifications of the quantification.

#### Parameterization of farmer’s behavior and biophysical environment

Farms and farmers: We synthesized different data sources to create a dataset with information on farm location, farm size, involvement in direct sales, presence/absence of small landscape elements, farmer age group, and the presence of a successor per individual farm of the case study area (Supplementary material, Table S2a). Farm addresses were coupled to a database with address coordinates (Kadaster 2020). A farm’s involvement in the direct sales was checked through a field (Zethof 2018) and Google Streetview inventory of direct sale outlets. Farm size (ha) was derived from the farm’s basic payment (€260 per hectare) as recorded in the public Common Agricultural Policy database (Rijksdienst voor Ondernemend Nederland 2020).

Maps of small landscape elements were combined and overlayed with the parcel map (Supplementary material, Table S2a) in ArcGIS 10.4, to identify whether each parcel contained, or was adjacent to, small landscape elements.

Because no map of parcels belonging to a certain farm is publicly available, we assigned parcels to farms based on proximity and used the constructed parcel map to calculate the percentage of farms with small landscape elements. We fed the farm size as derived from the Common Agricultural Policy database into a mass allocation in ScapeToad Cartogram and overlayed this with the parcel map, to couple parcels to farms (Zagaria et al. 2017). This was done separately for dairy and fruit farms, where orchards were coupled to fruit farms and grasslands and fodder parcels to dairy farms. The farms that receive a payment for agri-environmental measures were also classified as having small landscape elements.

Farms who receive payment for young farmers were classified as farms with a young (<40 years) owner. Furthermore, the farmer age distribution in the dairy and fruit sector and the average percentage of farmers with a successor was derived from the Farm Accountancy Data Network database (European Commission 2018) for the case study area as a whole (Supplementary material, Table S2a). Farm location was classified as close to built-up areas, remote, or located at an intermediate location. This was derived from the land cover map (Supplementary material, Table S2a). The distance was classified using natural breaks.

The uptake of efficiency (FoA2) measures was derived from the Farm Accountancy Data Network dataset for the Netherlands as a whole, for the farming types “specialist orchards – fruit” and “specialist milk”. The Farm Accountancy Data Network database (European Commission 2018) is the only accessible database that provides the required farm-level data, and was therefore used to calculate a proxy for the uptake of FoA2 SI measures. Based on (Weltin et al. 2018), we made a list of farm-level measures that affect resource use efficiency, knowledge management, and livestock fodder. We next checked the list of Farm Accountancy Data Network variables to find variables that directly provided indicators for the level of implementation of the SI measures, or could be used to calculate the level of implementation. This process was done by three experts individually and discussed afterwards, and resulted in a list of 12 measures (Supplementary material, Table S2b). After the measures were defined (Table S1), the uptake at farm level was calculated in R, using the package gmodels. We next identified for each measure if a farm did (1) or did not (0) adopt the measure, using the lower 10% quantile for each measure across all farms in the country as a threshold. Third, we counted how many FoA2 SI measures each farm adopted and calculated the average across all dairy farms and across all fruit producers separately. Fruit growers on average had 2.6 FoA2 measures implemented, dairy farms 4.3 measures.

In the interviews (section *Interviews*), stakeholders indicated that the intrinsic attitude of the farmer is more important than the influence of other actors upon deciding to adopt a measure. Respondents and workshop participants (section *Workshops*) estimated that ~5–10% of farmers are keen to try out innovations and about 10–20% lag behind. As the interview respondents and workshop participants did not provide explanatory factors for intrinsic motivation, we randomly classified 10% of farmers as intrinsically innovative.

Beyond intrinsic motivation, local conditions influence the uptake of SI measures: we found that farms with direct sales tend to be located closer to villages than farms without, which we parameterized as farms close to villages having a higher probability of taking up direct sales (see supplementary material 3). We also used farm size (in hectares) as an indicator for the likelihood of uptake of new measures, because the Farm Accountancy Data Network dataset shows that farmers who have SI measures in place tend to have larger farms (European Commission 2018). Furthermore, in the workshops, stakeholders indicated that particularly young farmers or farmers with a successor are the key innovators. We, therefore, assigned a higher probability of measure implementation to young farmers and farmers with a successor.

#### Parameterization of the social environment

The social environment of farmers was derived from the social network analysis and implemented in the model as a set of actors who provide advice to farmers about measure implementation, and a quantification of the probability that farmers adopt these. The role and importance of actors are clearly different for the different SI measures (Table 1). We used an initial ranking of the importance by two researchers that was confirmed by two stakeholders (Table 1) to quantify the role of the actors in farmers’ decision making. The highest-ranked actor was used to set a baseline value, the other actors, in rank order, provided a modifier to that (see supplementary material 3).

**Table 1:** Overview of actors and their importance in steering farmers’ attitude toward SI

Actor	Actor group			Ranks		
	Local/non-local	Market/government	Formal/informal	FoA1/3	FoA2	FoA4
Farmers	Local		Informal	4		1
Consumers	Local	Market	Informal	4		2
Retail/purchasers	External	Market	Formal	3	2	3
Province	Local	Government	Formal	1		4
National/EU government	External	Government	Formal	2	1	
Network	Local		Informal		3	
Farmer organizations	External		Informal		3	
Labels	External	Market	Informal	3	2	5

The “actor group” describes how actors are classified in the sensitivity analysis (section *Identification of enabling factors through sensitivity analysis*). The ranks indicate the order of importance of the actors for the different SI measures, where the most important actor is ranked as 1

FoA 1/3 small landscape elements: EU and national regulations are considered of prime importance for the establishment and maintenance of small landscape elements. The province specifies and funds a nature and agri-environmental measures plan, and is considered a key advisor. Secondly, incentives by labels and purchasers / retail that stimulate maintenance of small landscape elements were considered important. Furthermore, during our workshops and interviews, stakeholders indicated that contact with citizens, particularly through direct sales, triggers farmers to maintain or establish small landscape elements.

FoA2 efficiency measures: the uptake is considered to be primarily influenced by the national and EU legal framework; the obligation to adopt measures is considered the most important. Furthermore, purchasers and labels are perceived as key actors, although interviews indicated that retail organizations do not perceive themselves as frontrunners for improving sustainability in general. They aim to set standards that improve the base level but are feasible for many farmers. Advice from farm organizations and the network is considered highly relevant by the stakeholders. Consumers have a less profound, and indirect role.

FoA4 direct sales: Farmers’ intrinsic motivation is considered the prime driver for the uptake of direct sales, making them the key actor for uptake (Table 1). Secondly, uptake by consumers, and the resulting economic return, is considered essential. The survey among recreationists showed that 62% buy products at farm outlets more than twice a year. Fruit and fruit products comprise 75% of the purchases. Stakeholders contested the role of retailers in triggering direct sales. Workshop participants indicated that the increasingly strict attitude of retail triggers farmers toward alternative sales channels. Farmer organizations and the province play an advisory role. Labels help to initiate and advertise direct sales, but the workshop participants judged their outreach as low. Many (two-third) farmers seek advice from their networks, but this is not always adopted.

#### Identification of enabling factors through sensitivity analysis

To explore which actors and policy and regulatory context options are key enabling factors in the uptake of SI measures, we did a sensitivity analysis using the BBN. By doing four different sets of sensitivity runs, we inferred how the case study will evolve if specific groups of actors take a supportive or unsupportive role. The uptake levels of the outputs were recorded, and for the runs where all actors are assumed supportive or unsupportive, the output for each location-farm size-age combination was calculated and linked to the spatial data on farm and field distribution, for visualization and interpretation.

As a first set of sensitivity runs, we assumed all actors to take a supportive or unsupportive role. Next, we divided the actors into local actors that are located in, and directly linked to, the case study area, and external actors (Table 1). A third sensitivity analysis explored the role of formal vs. informal actors, in which formal actors include those who, according to the interview respondents, impose legal regulations or strict commercial quality standards. A fourth explored the role of government vs. market actors. Actors not classified in any of the groups were assigned a neutral attitude (Table 1).

## Results

### Regional Challenges and Potential Contribution of Sustainable Intensification

Stakeholders perceive pressure on space as a key issue, as revealed in increasing conflicts between recreation and farming. Furthermore, stakeholders see increasing pressures on water quality and biodiversity. Social and economic challenges include a perception of very high regulatory pressures that inhibit a level playing field and cause unfair competition with farmers in countries outside the EU; overproduction of agricultural products; and too low prices of crops and milk.

A desired future for the agricultural landscape in the case study area pivots around a stronger emphasis on regional integration of SI measures (Weltin et al. 2018), where policy incoherence is resolved and strong local marketing and value creation exists. Stakeholders expect and value the continuation of the current increase in efficiency (FoA2) measures. At the province level, the province envisions a trend toward mitigation and adaptation of climate change, better protection of biodiversity, increasing landscape quality and increasing use of the landscape for recreation. The agricultural sector is envisioned to support this by a transition toward sustainable and multifunctional agriculture with a focus on nature quality and landscape management and expansion of farm activities (Provincie Utrecht 2010). Workshop participants preferred strengthening of the small-scale, land-sharing landscape of the case study area, de-intensification, more organic farming, increased diversification, and a stronger mix of nature and farmland, that supports functional biodiversity. They prefer population growth to be accommodated in villages rather than sprawled.

### Network Analysis

Given stakeholder’s future preferences (section *Regional challenges and potential contribution of sustainable intensification*), the social network analysis focused on three tangible measures: implementation of small landscape elements (SLEs, FoA1/3), farm-level structural measures that improve efficiency (FoA2) such as reducing pesticide use (fruit) or optimizing grazing schedules (dairy) (full list of measures in supplementary material 2), and taking up direct sales (FoA4).

The social network analysis (see supplementary material 4) shows that farmers have the highest indegree across all topics. The high indegree and low outdegree of farmers regarding SLE’s and efficiency measures reflect the role of farmers as the implementers of measures, who should respond to the challenges identified by the stakeholders. On these topics, external actors including national and EU governance have more influence than the local actors that are directly related to the case study area and its local supply chain.

The network analysis on the uptake of direct sales shows a different picture, where farmers have a high outdegree, indegree, and centrality, suggesting a locally organized topic where farmers have agency. Consumers play a smaller role for all topics, where especially the moderate outdegree for direct sales is striking.

### Role of Actors in SI Uptake

**Fig. 5:**
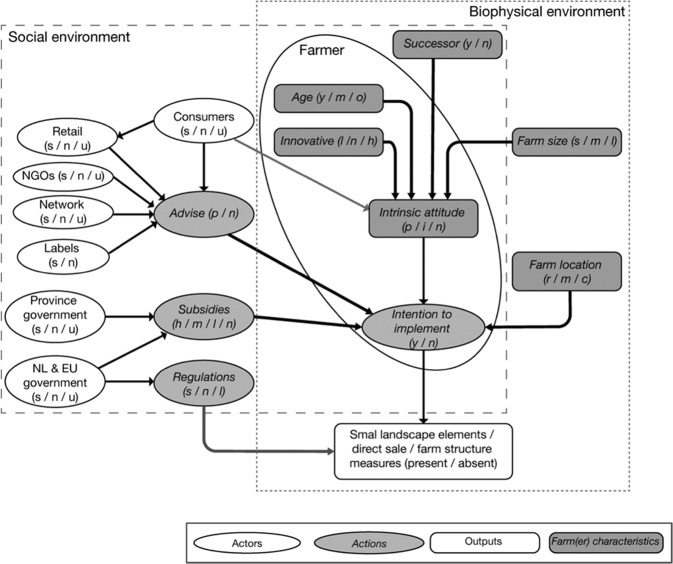
Structure of the BBN. Text between brackets indicates the potential states of the node: (s/n/u) supportive/neutral/unsupportive; (y/m/o) young/medium aged/old; (s/m/l) small/medium/large; (r/m/c) remote/moderate / close); (y/n) yes/no; (p/n) positive/negative. Node probability distributions in the baseline situation is given in Table 2

BBNs were constructed for the dairy and fruit farms separately (Fig. 5). Baseline conditions are shown in Table 2.

**Table 2:** Node probability distributions in the baseline situation

Node	Dairy			Fruit		
*4a. Actors*	Supportive	Neutral	Unsupportive	Supportive	Neutral	Unsupportive
Retail	33.3%	33.3%	33.3%	33.3%	33.3%	33.3%
NGOs	33.3%	33.3%	33.3%	33.3%	33.3%	33.3%
Network	33.3%	33.3%	33.3%	33.3%	33.3%	33.3%
Labels	50%	50%	n/a	50%	50%	n/a
Province	33.3%	33.3%	33.3%	33.3%	33.3%	33.3%
NL&EU	33.3%	33.3%	33.3%	33.3%	33.3%	33.3%
Consumers	19%	28%	53%	28%	37%	38%

*4b. Farm/farmer’s characteristics*	Dairy			Fruit		
Intrinsic attitude	Supportive: 45%	Neutral: 27%	Unsupportive: 28%	Supportive: 42%	Neutral: 29%	Unsupportive: 29%
Age	Young: 21%	Medium: 53%	Old: 26%	Young: 29%	Medium: 37%	Old: 34%
Innovative	Yes: 10%	Neutral: 75%	No: 15%	Yes: 10%	Neutral: 80%	No: 10%
Successor	Yes: 29%		No: 71%	Yes: 7%		No: 93%
Farm size	Small: 26%	Medium: 58%	Large: 17%	Small: 36%	Medium: 37%	Large: 27%
Farm location	Close: 24%	Medium: 42%	Remote: 34%	Close: 30%	Medium: 57%	Remote: 34%

*4c. Advice*	Dairy			Fruit		
FoA2 Efficiency	57%		43%	56%		44%
FoA1/3 Small landscape elements	52%		48%	52%		48%
FoA4 Direct sales	40%		60%	41%		59%
Subsidies	High: 64%	Medium: 16%	Low: 17%	High: 64%	Medium: 16%	Low: 17%
Regulations						
FoA2 Efficiency	High: 62%	Medium: 28%	None: 11%	High: 60%	Medium: 29%	None: 11%
FoA1/3 Small landscape elements	High: 61%	Medium: 28%	None: 11%	High: 61%	Medium: 28%	None: 10%

*4d. Intention to implement*	Dairy			Fruit		
	Positive	Negative		Positive	Negative	
FoA1/3 Small landscape elements	52%	48%		51%	49%	
FoA2 Efficiency	49%	51%		45%	55%	
FoA4 Direct sales	29%	71%		30%	70%	

*4e. Uptake*	Dairy			Fruit		
	Yes	No		Yes	No	
FoA1/3: Small landscape elements	36%	64%		35%	65%	
FoA2: Efficiency	53%	47%		40%	60%	
FoA4: Direct sales	20%	80%		22%	78%	

The BBNs of dairy and fruit show similar responses if the behavior of actors is modified in the sensitivity analysis.

For the uptake of *small landscape elements (FoA1/3)*, decreases can occur despite a supportive attitude of external actors (Table 3). The strongest increase in uptake is seen when all actors are supportive for dairy farmers, or with an unsupportive attitude of external actors combined with a supportive attitude of local actors for fruit growers. Changes in the attitude of market and government actors result in the strongest changes in uptake. Combined with the spatial distribution of farm type and size, actors can make the biggest difference along the Rhine and north of Langbroek (Fig. 4; for locations, see Fig. 2). This part of the case study area is dominated by medium-sized and larger dairy farms, which are the most likely to expand small landscape elements.

**Table 3:** Change in the adoption of output indicators in the sensitivity analysis cases including trade-offs and synergies and alignment with stakeholder’s vision on the future of the case study area

	Dairy				Fruit				Alignment with stakeholder vision
	T/S^a^	FoA1/3	FoA2	FoA4	T/S^a^	FoA1/3	FoA2	FoA4	
*Input (% adoption)*		29%	45%	11%		14%	33%	9%	
*Impact of external (E)* vs*. local (L) actors (% change)*									
E supportive, L neutral	S−	−31%	−9%	−9%	T	−29%	20%	5%	No
E unsupportive, L supportive	S−	−19%	−6%	−6%	S+	***43%***	25%	*59%*	No
E supportive, L unsupportive	S+	3%	17%	17%	T	6%	53*%*	−32%	No
E supportive, L neutral	S+	*17%*	*19%*	*19%*	T	20%	*58%*	−5%	No
*Impact of market (M)* vs. *government (G) actors (% change)*									
G unsupportive, M neutral	T	−33%	−4%	5%	T	−31%	25%	0%	No
G unsupportive, M supportive	T	−33%	−2%	*30%*	T	−34%	30%	*23%*	No
G supportive, M unsupportive	T	*17*%	11%	−5%	T	*17%*	48%	−9%	No
G supportive, M neutral	S+	14%	*15%*	5%	T	*17%*	*50%*	−9%	Almost
*Impact of formal (F)* vs. *informal (I) actors (% change)*									
I unsupportive, F neutral	T	−11%	2%	−30%	T	−9%	35%	−32%	No
I unsupportive, F supportive	T	3%	9%	−35%	T	6%	45%	−36%	No
I supportive, F unsupportive	T	−17%	0%	***70%***	T	−14%	33%	***64%***	No
I supportive, F neutral	S+	*17%*	*15%*	60%	S+	*20%*	*53%*	55%	Yes
All actors unsupportive	S−	−39%	−13%	−20%	T	−40%	15%	−23%	No
All actors supportive	S+	**33%**	**23%**	55%	S+	37%	**63%**	50%	Yes

Percentages in bold indicate the strongest increase per output indicator; italic percentages indicate the strongest increase of each output indicator within each actor grouping

^a^Trade-offs or synergies. S−: all indicators decrease; S+: all indicators increase; T: some of the indicators increase and some decrease

The uptake of *efficiency measures (FoA2)* is primarily influenced by external actors (Table 3). For both dairy and fruit, the highest change in uptake is seen with all actors supporting uptake, followed by a supportive attitude of external actors. For fruit, also the sensitivity analysis with a positive role of the informal actors results in a strong increase. Overall, increases in the fruit sector are stronger than in the dairy sector, where decreases of FoA2 uptake might occur under a few conditions, namely a negative role of external or government actors.

**Fig. 6:**
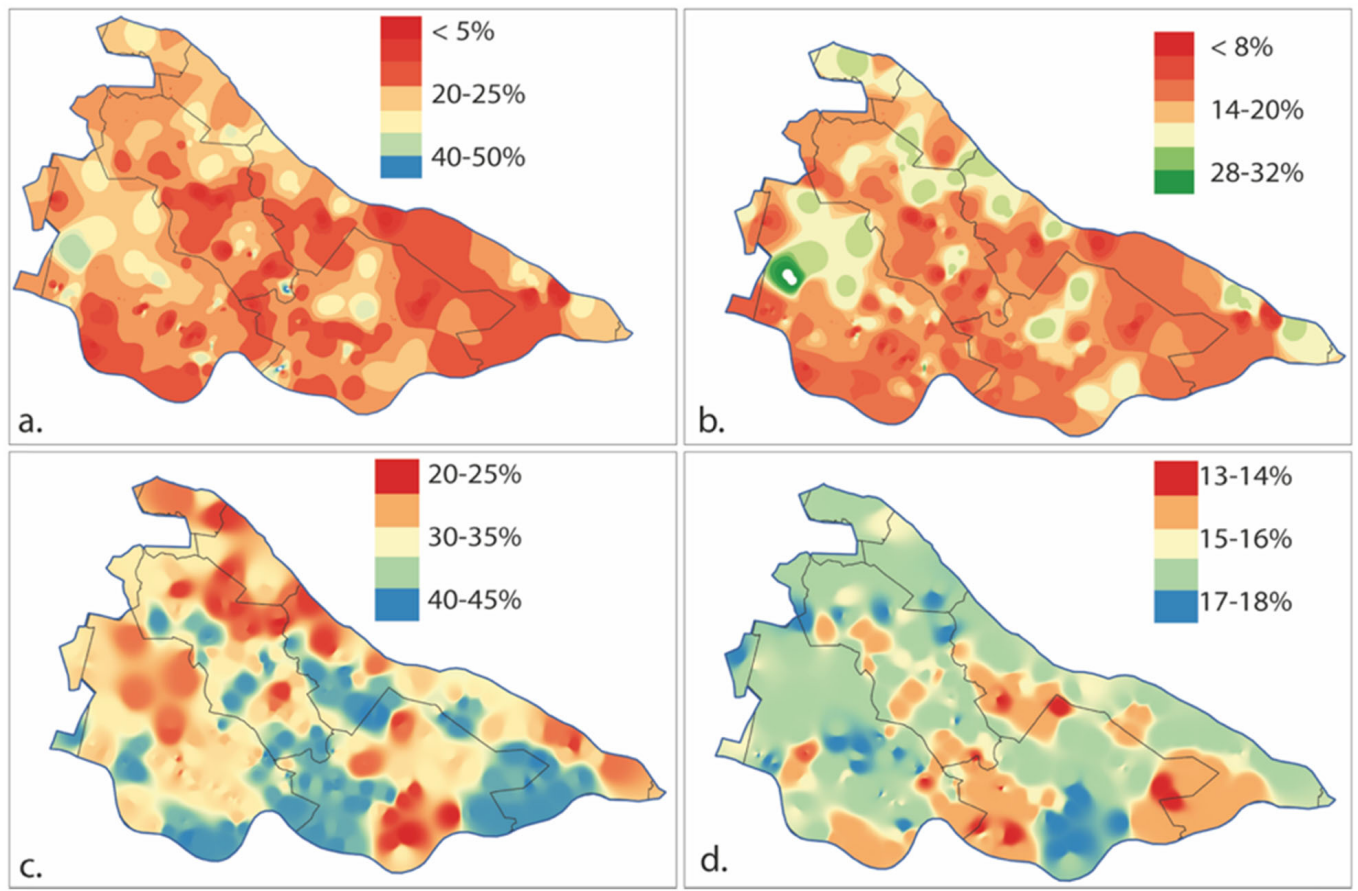
Top: probability that a farm is involved in direct sale (**a**) or has small landscape elements (**b**). Bottom: Impact of actors on uptake of (**c**) direct sale and (**d**) small landscape elements, quantified as the difference between a scenario where all actors have a supportive attitude and a scenario where all actors have an unsupportive attitude

The highest increase in *direct sales (FoA4)* uptake occurs with a positive attitude of informal actors such as consumers and farmers (Table 1), and a negative attitude of formal actors such as purchasers (Table 1; Table 3). In the different sensitivity explorations, an important role for consumers and farmers appears, i.e., local, informal actors who are related to the supply chain. Changing the support of informal vs. formal actors yields the largest sensitivity, where a full supportive attitude of all informal actors can increase the uptake by 70% and a full unsupportive attitude can decrease uptake by 35% (Table 3). Combined with the spatial distribution of farm type and size, actors can make the biggest difference around Wijk bij Duurstede (for locations, see Fig. 2) and in scattered pockets in the west of the case study area (Fig. 6). This is particularly due to the high accessibility of these parts of the case study area.

Overall, most sensitivity runs result in a trade-off. When varying the support of government and market actors, an unsupportive attitude of government decreased direct sale and FoA2 measures and increased SLEs (FoA1/3). For the analysis of the role of informal and formal actors, an increase of direct sale (FoA4) uptake depended on a supportive role of the informal actors (Table 3). For the dairy sector, assigning an unsupportive attitude to all actors provides a decrease in all outputs. Synergies are seen in the simulations with all actors having a supportive attitude and in simulations with positive external actors, positive government actors, and positive informal actors (Table 3). For the fruit sector in the case study area, synergies are seen in the simulations with all actors having a supportive attitude, in the simulation where local actors are supportive while external actors oppose, and upon a supportive attitude of informal actors (Table 3).

Only the sensitivity analyses where all actors have a supportive attitude and where informal actors are supportive and not opposed by formal actors result in an increase of all output indicators for both farm types, which can be considered aligned with the stakeholders’ preferences (section *Regional challenges and potential contribution of sustainable intensification*). The simulations with a supportive role of external or government actors almost align with the stakeholders’ future preferences but result in a continuation or small decrease in direct sales (FoA4) uptake for one of the farm types.

## Discussion

### Enabling Factors for Sustainable Intensification

Through the sensitivity analysis, we identified enabling factors for the uptake of three SI measures. A comparison with three other case studies from the same group of projects project showed that the Kromme Rijn area has a dense network (Schmidt et al. 2018). While dense networks might increase the chance that shared values emerge among stakeholders (Rowley 1997), in peri-urban areas like Kromme Rijn values of different stakeholders are intrinsically different. Also, many connections in our network are asymmetrical. This might place farmers in a subordinate or compromising role in the network, in which conflicting positions of other stakeholders might lead to inaction (Rowley 1997).

For *direct sales*, the BBN exploration suggested that particularly farmers and additionally consumers are enabling actors. As central actors in a dense network, they enable a compromise between different influential stakeholders (Rowley 1997). The external and formal actors, including retail and the national government, primarily steer toward production efficiency, and form a barrier. An observed high increase in direct sale uptake under a negative attitude of formal actors might also reflect an observation by our stakeholders that the attitude of supermarkets with regard to prices and regulations served as a trigger for farmers to seek alternative sales channels. Recent research showed that this sentiment is shared by many Dutch farmers, as the competition for consumers by supermarkets and other market parties in the food chain gives a continuous pressure on prices, also in the agricultural chains (Baltussen et al. 2018). Low satisfaction with retail also triggered farmers to adopt direct sales in a case study in Greece (Tselempis et al. 2020), potentially reflecting a similar process. Tselempis et al. (2020) also highlighted how interaction between consumers and farmers triggered farmers who perceived consumer interest in local food to start direct sales. This interaction particularly takes place at easily accessible locations (Fig. 4) and, as “I just passed by the direct sales outlet” was an important reason for consumers to purchase at a farm according to our survey (Komossa et al. 2019), these locations seem key for initiating the interaction between consumers and farmers.

For *farm-level efficiency* measures, formal actors are more pronounced triggers. Although Thorlakson et al. (2018) suggest that business environmental standards can stimulate farm sustainability, our study suggests that while a supportive attitude of market actors increases the uptake of FoA2 measures, formal actors seem essential to maximize uptake. Stakeholders state that the external market actors stimulate incremental steps to increase environmental standards, (e.g., slightly reducing pesticide residual thresholds), while formal actors tend to set more transformative targets. This is reflected in e.g., our interviews with retail organizations. This can also be seen in light of the different motives that market parties usually have toward the stimulation of sustainable measures compared to formal actors. Research in the Netherlands, for instance, found that most motives where focused on reputation management, safeguarding long-term supply of products and opportunities for new consumer markets (De Krom and Prins 2019). The lower centrality of the farmers on this topic might render a “subordinate” role, where such triggers by more influential actors are readily taken up (Rowley 1997). (Rowley 1997). An important factor here is the expectation that (part of) these measures will have an obligation to adopt in the future.

For *small landscape elements*, a strong trigger by local actors can compensate unsupportive external actors (Table 3) for fruit farmers, but overall, commitment of a broad group of actors yields the strongest effects. Actors at national, regional and local scale related to the implementation of the CAP obviously play an important role (Hauck et al. 2016). Furthermore, farmers mention that learning about consumer’s positive perceptions of small landscape elements through direct contact with consumers upon direct sales triggers establishing and maintaining small landscape elements. Such a role of local food networks in fostering biodiversity is also found by (Simoncini 2015).

Stakeholders preferred a future with strengthened regional value chains, a clear separation between the urban and rural landscape, and land sharing within the rural landscape mainly leading to diverse mix of different farming systems with natural elements. Stakeholders acknowledged the relevance of land-sparing, but considered it unfit for their situation. Similar visions are found in case studies in England and Ontario (Marr and Howley 2018). This vision is compromised by the difficulty of avoiding trade-offs between different SI fields of action, which often emerge upon opposing positions of different groups of actors (Table 3). For instance, while direct consumer contact through direct sales can stimulate farmers to establish small landscape elements, a lack of support for general sustainability goals by external actors triggers both uptake of direct sales as well as scale enlargement that might decrease maintenance of small landscape elements. However, the desired separation between urban and rural regions might protect peri-urban open space from transformation, and zoning policies and wider, binding inclusion of the ecosystem services concept in governance provides a tangible course of action for achieving this (Spyra et al. 2021).

The overall level of support for SI by the actor network has the strongest effect on SI uptake for fruit farms at easily accessible locations (Fig. 4). Consistent with Foguesatto et al. (2020) and Lange et al. (2013), farms with direct sales tend to be located closer to residential areas than farms without. Locations, where actors can make a difference in the northern and eastern part of the study area, are areas dominated by mid-sized or larger dairy farms, which are most likely to adopt small landscape elements when supported to do so. As these areas coincide with locations highly appreciated by recreationists in the case study area (Tieskens et al. 2018), expansion of green infrastructure might further stimulate recreation and with that short value chains.

### Limitations of the Methodology

Stakeholder participation in studies on landscape sustainability reveals knowledge otherwise difficult to disclose, and adds realism and legitimacy to proposed solutions (Rounsevell and Metzger 2010, Mathur et al. 2008). This was obvious in our study, where the broad insights from stakeholders throughout the process aided understanding the case study. Risks and difficulties upon stakeholder participation include increasing attention for stakeholder participation at funders, potentially resulting in over-asking, followed by stakeholder fatigue (Hagemann et al. 2020). Furthermore, our study was embedded in research projects with pre-established aims, objectives, and deliverables, that limited flexibility toward stakeholder knowledge that emerged in the participatory process. Funding for transdisciplinary research projects needs to allow such flexibility.

The BBN analysis allowed combining scattered and varied information in a quantitative analysis that gives insight into the comparative role of different actors. BBNs are increasingly used for analyzing complex, ambiguous systems (Salliou et al. 2017). However, quantification of the social network analysis as well as the BBN has a component of subjectivity that can lead to uncertainties in the outcomes. A concept of the BBN was discussed with stakeholders, leading to adaptations that have reduced uncertainties. In the sensitivity analysis, we deliberately explored extreme future developments, to delineate the future option space.

The wide variety of data for quantification of our farm systems and drivers for change might have introduced inconsistencies. Parameterization of farm size, age, and FoA2 uptake was based on data from the Farm Accountancy Data Network (European Commission 2018), potentially introducing a bias toward larger farms.

### Implications

In this study, we adopted a broad perspective on SI that addresses the whole food system, combined with modelling interaction among actors, to provide a novel perspective on potential triggers and barriers to sustainability. The multiple demands on our case study call for ways to integrate the delivery of multiple public goods in a farm’s structure. However, in line with Zscheischler et al. (2019), our model-based exploration of the interaction among the stakeholders shows how different priorities of stakeholders regarding sustainable agriculture can hinder progress. We found that trade-offs between different SI measures were difficult to avoid. Only if all actors are aligned, progress on all indicators was expected. In particular, on-the-ground commitment was essential. However, two out of three sensitivity analyses that (almost) match the stakeholder vision (Table 3) have a supportive role of government actors. This is consistent with Martin-Lopez et al. (2019) who showed that power relations among actors tend to be asymmetrical, with the power at larger-scale stakeholders and the dependence of the local landscape with local actors. A better understanding of actor interactions and their social network can provide insight into policy implementation outcomes and the local governance capacity (Ptak et al. 2022). Our results also show that the influence of social factors should receive enough attention in policy design processes, as a narrow focus on economic motivators can lead to misalignment of policy outcomes (Mills et al. 2016; Ptak et al. 2022).

Consistent with Simoncini (2015), our study shows that direct sales initiatives can cascade into increased provision of public goods. Accommodating direct sales might thus support the delivery of public goods demanded by peri-urban stakeholders, with the increased involvement of consumers in short chains triggered by the early COVID-19 pandemic potentially leading to continuation of willingness to support locals in the medium to long term (Hobbs 2020). In addition, specific policies that target compact settlements and aim at protection of the natural environment will help support the provision of public goods from open spaces in peri-urban areas (Spyra et al. 2021). This requires a consistent, result-oriented policy bundle that is flexible and adaptive (Spyra et al. 2021), where a fair and open deliberation between stakeholders at different scales (Martin-Lopez et al. 2019) contributes to a fair distribution of all the trade-offs.

The complexity and variability of stakes in peri-urban areas makes similar trade-offs as in our case study likely across northwest Europe. While in other case studies, other public good combinations might be demanded and other SI measures might work best, the complexity of the planning challenges of peri-urban areas (Geneletti et al. 2017) is likely to render trade-offs. The SI framework by Weltin et al. (2018) provides guidance for choosing which dimensions are relevant in a specific case study. Urban food strategies as a new governance approach (Doernberg et al. 2019) might help overcome the policy incoherence between food, agriculture, and environmental policy (Galli et al. 2020). This requires knowledge sharing between the different actors involved (Schaller et al. 2018) and alignment of local and larger-scale targets (Wolff et al. 2020). Such a food strategy might include support for local food chains that support economic farm resilience, cascade into the uptake of other measures that target sustainability, and support recreation. To consolidate such changes and foster further change, context-specific contracts that monetize public good provision between land managers on the one hand and government/supply chain actors on the other hand might both stimulate the frontrunners toward transformation and increase the sustainability base level for the broader farmer community (Tal 2018).

## Conclusions

We explored the impact of different groups of actors on the uptake of three SI measures and evaluated how uptake aligns with a stakeholder vision toward a sustainable future of a peri-urban case study area in the Netherlands. The stakeholders preferred a land-sharing landscape with low-intensity agriculture that takes stock of the latest technologies and preferred a transition to a strong local supply chain. We, therefore, investigated the uptake of SI measures targeting these preferences: direct sales, efficiency increases, and small landscape elements. We found that trade-offs between different SI measures were difficult to avoid. Only if all actors were aligned, progress on all indicators was expected. In particular, an on-the-ground commitment was essential.

## Supplementary information

Supplementary Information
