# Supplementary material for: The Role of Different Types of Actors In The Future of Sustainable Agriculture In a Dutch Peri-urban Area
Source: Environ Manage. 2022 May 4;70(3):401–19. doi: 10.1007/s00267-022-01654-3 (PMC9065672; doi:10.1007/s00267-022-01654-3)
Supplement: Supplementary file 1 — Supplementary Information [file 267_2022_1654_MOESM1_ESM.docx]

**Supplementary material**

**Supplementary material 1: Workshop participants**

*Workshop 1: introduction to region and its spatial challenges*

*Workshops 2a: Status and outlook for SI in the study area*

*Workshop 2b: Participatory development of alternative land use futures*

*Workshop 3: Integrated vision on the future of the region*

Each ID reflects an individual workshop participant. An X indicates that this participant attended the workshop. Participants are sorted randomly. Workshop participants listed in italic were also included in the interview phase.

| **ID** | **Organization type / occupation** | **1** | **2a** | **2b** | **3** |
| --- | --- | --- | --- | --- | --- |
| 1 | Regional leisure and tourism organization |  | X | X |  |
| 2 | Estate manager |  |  | X |  |
| *3* | *Utrecht province* |  |  |  | X |
| 4 | National forest and nature management organization |  | X | X |  |
| 5 | Fruit grower |  | X |  |  |
| 6 | Nature collective Utrecht |  | X |  | X |
| 7 | Representative of the municipalities | X |  |  | X |
| 8 | Regional rural development cooperative |  | X | X |  |
| 9 | Province heritage and landscape organization |  | X |  |  |
| 10 | Dutch Land Owners Organization |  |  |  | X |
| 11 | Utrecht province |  | X | X | X |
| *12* | *Fruit grower* |  | X | X |  |
| 13 | Scientist | X |  |  |  |
| 14 | Netherlands Agricultural Association |  | X |  | X |
| 15 | Province heritage organization | X |  |  |  |
| *16* | *Fruit processor* |  | X |  |  |
| 17 | Regional rural development cooperative |  |  |  | X |
| 18 | Utrecht province | X | X |  | X |
| 19 | Netherlands Agricultural Association | X |  |  |  |
| 20 | Fruit producer |  | X |  |  |
| *21* | *Livestock farmer* |  | X | X | X |
| 22 | Representative of the municipalities | X |  |  |  |
| *23* | *Province nature and environment NGO* | X |  |  |  |
| 24 | Municipality officer |  | X |  |  |
| 25 | Province heritage and landscape organization | X |  |  |  |

***Supplementary material 2: Parameterization of farmer’s biophysical environment and SI uptake***

*Table S2a: Overview of input data to characterize farm and farmer characteristics*

| Topic | Data description | Reference |
| --- | --- | --- |
| Farm location and main type | | |
| Public CAP database | Database of 2019 CAP subsidies to companies and persons, specified by scheme (agri-environment measures, young farmers) | (Rijksdienst voor Ondernemend Nederland, 2020) |
| Organic farms | Online map and list of organic farms | (Malek et al., 2019) |
| Livestock farm database | Map and list of livestock farms | (Bij12, 2019) |
|  | | |
| Farm characteristics | | |
| Parcels | Agricultural parcel map 2018 | (Rijksdienst voor Ondernemend Nederland, 2019) |
| Small landscape elements | Point, line, and area small landscape elements in Utrecht province | (Provincie Utrecht, 2019) |
| Land cover / land use | Detailed land cover / land use map of the Kromme Rijn Region | (Verhagen et al., 2018) |
| Farm type, farmer age, uptake of SI measures | Farm Accountancy Data Network (FADN) | (European Commission, 2018) |

*Table S2b. List of measures indicative for the uptake of farm-level structure (FoA2) SI measures.*

| Measure | FADN indicator - description | FADN indicator - Code | Farm type |
| --- | --- | --- | --- |
| Reduction of fertilizer | Costs for purchasing fertilizers (in €) | SE295 | Both |
| Irrigation | UAA under irrigation (% of total UAA) | A40, SE025 | Fruit growers |
| (Green) manure and compost | Area of green legumes (% of total UAA) | K328AA, SE025 | Both |
| Crop residue use | Production of straw, beet tops, and other by products (tonnes) | K299QQ, K300QQ, K301QQ, K304QQ | Fruit growers |
| Closed resource cycles on farm | Livestock unit density (LSU/ha) | Or SE080, SE085, SE090, SE095, SE100, SE105, SE025 | Both |
| Planning of labour input | Annual Worker Units per UAA (ha) or LSU | SE010, SE025, SE080, SE131 | Both |
| Farm size enlargement | in ha | SE025, SE010 | Both |
| Knowledge as input in the production process | Support to advisory services (in €) | JC835 | Both |
| Management of soil functions | Area under fallow, green manure, deep rooting crops, or permanent grassland (% of total UAA); organic farming (Y/N). | K145AA, K328AA, K329AA, K360AA, K361AA, K147AA, K150AA, K151AA, K172AA, K173AA, K314AA, K315AA, K316AA, K330AA, A32 | Both |
| Protein sources | Legume fodder crops (% of total fodder crops) | K144QQ, K328QQ, K329QQ, K147QQ, K150QQ, K151QQ, K172QQ, K326QQ, K327QQ | Dairy |
| Efficient use of all farm inputs | Input-output ratio (in €) | SE132D & SE132N | Both |
| Efficient fuel use | Energy use (€) per ha UAA or LSU | SE345, SE025, SE080 | Both |

***Supplementary material 3: Conditional probability tables***

Based on the network analysis and the insights gained from the stakeholder workshops, we ranked the actors or environmental context variables for their importance in influencing a node, and estimated the relative importance of the different inputs into each CPT. Next, we used the most important input to set baseline values for each CPT (indicated in bold in Tables S3a-S3j) and used the importance of the other input to set modifiers.

Figure S3 shows the structure of the BBN and indicates the position of the different CPTs. Tables S3a-3n show the Conditional Probability Tables.


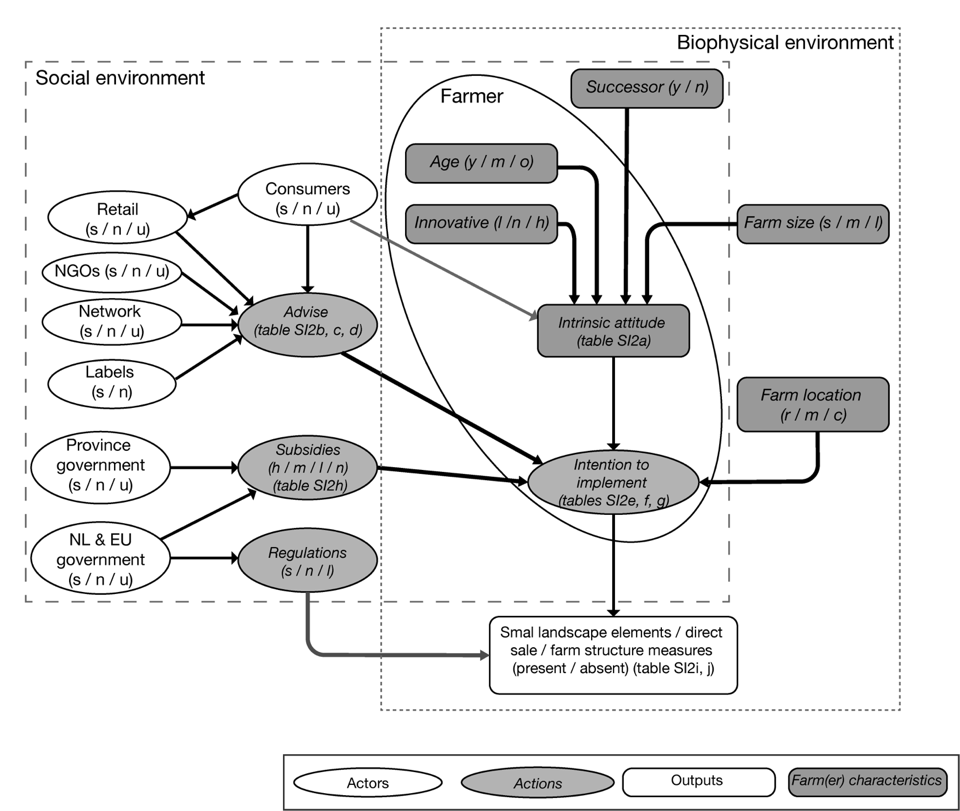


*Figure S3: structure of the BBN and linkage to CPTs.*

| **Table S3a. Probability a farmer has a positive intrinsic attitude towards SI implementation.** Probability of a neutral or negative attitude is (1-p_positive_)/2. | |
| --- | --- |
| **Innovativeness** | **high: 0.6; moderate: 0.45; low: 0.3** |
| Age / successor | young or moderate with successor: +0.1; old with successor: +0.05; moderate without successor: 0; old without successor: -0.15 |
| Farm size | large: +0.05; medium: 0; small: -0.05 |

| **Table S3b. Probability of a positive advice on FoA2 farm structure measures.** Probability of a neutral or negative attitude is (1-p_positive_)/2. | |
| --- | --- |
| **Retail** | **supportive: 0.6; neutral: 0.5; unsupportive: 0.4** |
| Labels | supportive: +0.15; neutral: 0 |
| Farm organizations | supportive: +0.05; neutral: 0; unsupportive: -0.05 |
| Environmental organizations | supportive: +0.05; neutral: 0; unsupportive: -0.05 |
| Network | supportive: +0.05; neutral: 0; unsupportive: -0.05 |

| **Table S3c. Probability of a positive advice on FoA1/3 small landscape elements.** Probability of a neutral or negative attitude is (1-p_positive_)/2. | |
| --- | --- |
| **Environmental organizations** | **supportive: 0.7; neutral: 0.4; unsupportive: 0.1** |
| Retail | supportive: +0.1; neutral: 0; unsupportive: -0.1 |
| farm organizations | supportive: +0.1; neutral: 0; unsupportive: -0.1 |
| Province | supportive: +0.05; neutral: 0; unsupportive: -0.05 |
| Network | supportive: +0.05; neutral: 0; unsupportive: -0.05 |

| **Table S3d. Probability of a positive advice on FoA4 direct sale.** Probability of a neutral or negative attitude is (1-p_positive_)/2. | |
| --- | --- |
| **Retail** | **supportive: 0.3; neutral: 0.45; unsupportive: 0.6** |
| Labels | supportive: +0.05; neutral: -0.05 |
| Farm organizations | supportive: +0.075; neutral: 0; unsupportive: -0.075 |
| Consumers | supportive: +0.05; neutral: 0; unsupportive: -0.05 |
| Province | supportive: +0.1; neutral: 0; unsupportive: -0.1 |

| **Table S3e. Probability of a positive farmer's intention to implement FoA2 farm structure measures.** Probability of a negative intention is (1-p_positive_). | |
| --- | --- |
| **Farmer** | **high: 0.65; moderate: 0.5; low: 0.35** |
| Advise | positive: +0.2; neutral: 0; negative: -0.2 |
| Direct sale | yes: +0.1; no: -0.1 |

| **Table S3f. Probability of a positive farmer's intention to implement FoA1/3 direct sale.** Probability of a negative intention is (1-p_positive_). | |
| --- | --- |
| **Farmer** | **high: 0.7; moderate: 0.5; low: 0.2** |
| Subsidies | high: +0.1; moderate: 0.05; low: 0 |
| Advise | positive: +0.05; neutral: 0; negative: -0.05 |
| Direct sale | yes: +0.1; no: -0.1 |

| **Table S3g. Probability of a positive farmer's intention to implement FoA4 direct sale.** Probability of a negative intention is (1-p_positive_). | |
| --- | --- |
| **Farmer** | **high: 0.5; moderate: 0.35; low: 0.2** |
| Advise | positive: +0.05; neutral: 0; negative: -0.05 |
| Location | close: +0.1; moderate: 0; remote: -0.2 |
| Consumers | supportive: +0.1; neutral: 0; unsupportive: -0.05 |

| **Table S3h. Probability of high subsidies for SI implementation.** Probability of a neutral or low subsidyis (1-p_positive_)/2. | |
| --- | --- |
| **External** | **supportive: +0.8; neutral: 0.45; unsupportive: 0.3** |
| Local | supportive: +0.2; neutral: 0.1; unsupportive: 0 |

| **Table S3i. Probability of implementation of FoA2 measures.** Probability of a no implementation is (1-p_positive_). | |
| --- | --- |
| **Regulations** | **strict: 0.7; neutral: 0.6; loose: 0.5** |
| Intention | positive: +0.05; neutral: 0; negative: -0.2 |

| **Table S3j. Probability of implementation of small landscape elements (FoA1/3).** Probability of a no implementation is (1-p_positive_). | |
| --- | --- |
| **Regulations** | **strict: 0.5; neutral: 0.3; loose: 0.1** |
| Intention | positive: +0.25; neutral: 0; negative: -0.4 |

***Supplementary material 4: Results of Social Network Analysis***

*Table S1: Social network indicators*

|  | FoA1/3: Small landscape elements | | | FoA2: Efficiency | |  | FoA4: Direct sale | |  |
| --- | --- | --- | --- | --- | --- | --- | --- | --- | --- |
| Actor | Weighted indegree | Weighted outdegree | Centrality | Weighted indegree | Weighted outdegree | Centrality | Weighted indegree | Weighted outdegree | Centrality |
| Local farmers | 22.00 | 4.00 | 0.25 | 25.00 | 5.00 | 0.47 | 15.00 | 21.00 | 1.00 |
| Local value chain initiatives | 12.33 | 4.67 | 0.27 | 7.50 | 3.17 | 0.42 | 7.33 | 10.67 | 0.67 |
| Retail | 6.00 | 8.33 | 0.54 | 10.33 | 9.17 | 0.59 | 1.67 | 1.67 | 0.20 |
| Farmer's organization | 13.00 | 10.00 | 0.53 | 11.00 | 9.50 | 0.62 | 6.00 | 9.00 | 0.70 |
| EU governance | 5.00 | 25.00 | 0.79 | 4.50 | 22.00 | 0.81 | 9.00 | 1.00 | 0.33 |
| National governance | 11.00 | 18.00 | 0.68 | 8.00 | 17.50 | 0.76 | 9.00 | 4.00 | 0.47 |
| Province | 7.50 | 6.50 | 0.39 | 9.25 | 6.25 | 0.52 | 4.00 | 4.50 | 0.35 |
| International market | 6.00 | 13.00 | 0.62 | 5.00 | 10.00 | 0.64 | 2.00 | 0.00 | 0.00 |
| Landscape NGOs | 8.50 | 17.00 | 0.68 | 6.50 | 10.75 | 0.68 | 5.50 | 0.00 | 0.00 |
| Other NGOs | 5.50 | 13.00 | 0.68 | 6.50 | 9.25 | 0.54 | 0.00 | 0.00 | 0.00 |
| Consumers | 5.00 | 5.00 | 0.35 | 8.50 | 4.50 | 0.49 | 10.00 | 8.00 | 0.61 |

**References**

Bij12 (2019) Bestand Veehouderijbedrijven. Bij12, Utrecht. 2018: June 2016. <https://data.openstate.eu/dataset/bestand-veehouderijbedrijven/resource/aa8b3b1e-6e3e-40a7-88b6-ec65d814f01e>

European Commission (2018) Farm Accounting Data Network An A to Z of methodology, version 02/07/2018. 2015. <https://ec.europa.eu/agriculture/rica/pdf/site_en.pdf>

Malek, Ž., Tieskens, K.F., Verburg, P.H. (2019) Explaining the global spatial distribution of organic crop producers. Agricultural Systems 176, 102680.

Provincie Utrecht (2019) Landschapsverordening. 10 October 2020. <http://geo-point.provincie-utrecht.nl/pages/open-data>

Rijksdienst voor Ondernemend Nederland (2019) Basisregistratie Gewaspercelen. Kadaster, 2018. <https://www.nationaalgeoregister.nl/geonetwork/srv/dut/catalog.search#/metadata/b812a145-b4fe-4331-8dc6-d914327a87ff?tab=general>

Rijksdienst voor Ondernemend Nederland (2020) Openbaarmaking Europese subsidiegegevens (disclosure of European subsidies). Producer, Version: Date of Collection.<https://mijn.rvo.nl/openbaarmaking-europese-subsidiegegevens>

Verhagen, W., van der Zanden, E.H., Strauch, M., van Teeffelen, A.J.A., Verburg, P.H. (2018) Optimizing the allocation of agri-environment measures to navigate the trade-offs between ecosystem services, biodiversity and agricultural production. Environmental Science & Policy 84, 186-196.
